# Unusual Presentation of a Severe COVID-19 Case With Axillary Artery Thrombosis

**DOI:** 10.7759/cureus.15036

**Published:** 2021-05-15

**Authors:** Fadi S Aljamaan

**Affiliations:** 1 Critical Care Department, King Saud University, Riyadh, SAU

**Keywords:** covid, thrombosis, critical care, arterial, embolectomy, axillary artery, hypercoagulability, anticoagulation

## Abstract

The coronavirus disease 2019 (COVID-19) pandemic has been evolving since early 2020 with high critical care mortality and morbidity. High mortality rates have been linked largely to respiratory failure. Hypercoagulability status induced by the massive inflammatory storm has led to a high rate of thrombotic events, whether arterial or venous, contributing to COVID-19 mortality especially in critically ill patients. Thrombotic events might be the presenting feature of the disease or might happen during hospitalization. In this case report, we describe a case of a 67-year-old male with severe COVID-19 pneumonia who was found on presentation to have left axillary artery thrombosis requiring embolectomy; the case was managed successfully. We reviewed the pathophysiology of hypercoagulability associated with COVID-19, clinical implications, and most recent treatment recommendations.

## Introduction

Coronavirus disease 2019 (COVID-19), as many other infectious disease, has shown a different range of disease severity, tending to cause severe respiratory failure and multiorgan failure especially in the elderly and those with comorbidities. Thrombotic events have been described early in the pandemic, especially in patients with severe pneumonia and having cardiovascular risk factors [1]. The disease has been associated with an increased risk of venous and arterial thrombosis in multiple studies [2,3], ranging from 4% up to 60%. The highest incidence rates were reported involving deep vein thrombosis (DVT) and pulmonary embolism (PE) more than arterial thrombosis and mostly in critically ill patients [4-7], while arterial thrombosis events that have been described were ischemic strokes, limb ischemia, mesenteric ischemia, and others [8-10].

We report a case of a 67-year-old male with severe COVID-19 who was diagnosed with severe adult respiratory distress syndrome (ARDS) and was found to have left axillary artery thrombosis causing limb ischemia. Initially, he was managed with a trial of endovascular thrombus retrieval and thrombolysis that failed and then with open embolectomy that was successful.

## Case presentation

**Figure 1:**
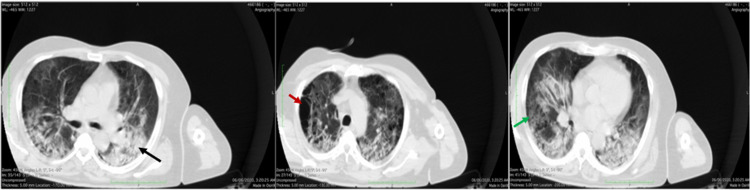
CT chest on day 1 showing baseline emphysematous changes (red arrow) and ground-glass opacities (green arrow) with consolidative changes (black arrow) involving both lungs, mainly dependent areas.

A 67-year-old male, who is a heavy smoker and known to have hypertension and benign prostate hypertrophy on treatment, presented to the emergency room on June 4, 2020, with a three-day history of cough, fever, and difficulty in breathing. He was admitted as a suspected case of COVID-19 for investigation and management. CT of the chest showed evidence of emphysema, patches of consolidation, and ground-glass opacities (Figure 1).

On initial evaluation, he had the following vitals: blood pressure of 140/80 mm Hg, heart rate of 80 beats/minute, respiratory rate of 30 breaths/minute, and oxygen saturation of 80% on room air. Due to significant hypoxia and labored breathing, he was intubated immediately and started on mechanical ventilation with FiO_2_ 50%, lung-protective strategy with ARDS protocol, and positive end-expiratory pressure (PEEP) recruitment. He received azithromycin 500 mg intravenous (IV) once daily (OD), ceftriaxone 2 gm IV OD, and methylprednisolone 40 mg IV twice daily (BID); enoxaparin 60 mg subcutaneously (SC) BID (full anticaogulation) was started from admission, which was a standard of care in our institution for severe COVID-19 disease; and antibiotics were switched after two days to levofloxacin 750 mg IV OD due to clinical worsening.

Baseline investigations showed D-dimer of >5 mcg /mL (normal range: 0 to <0.5 mcg), CRP of >120 mg/L (normal range: <5 mg/L), creatinine of 1.25 mg/dL (normal range: 0.6-1.2 mg/dL), WBC of 11,000 cells/mcL, lymphocytes of 1,820 cells/mcL, neutrophils of 8,440 cells/mcL, platelets of 342,000/mcL, and hemoglobin of 13.8 gm/L.

**Figure 2:**
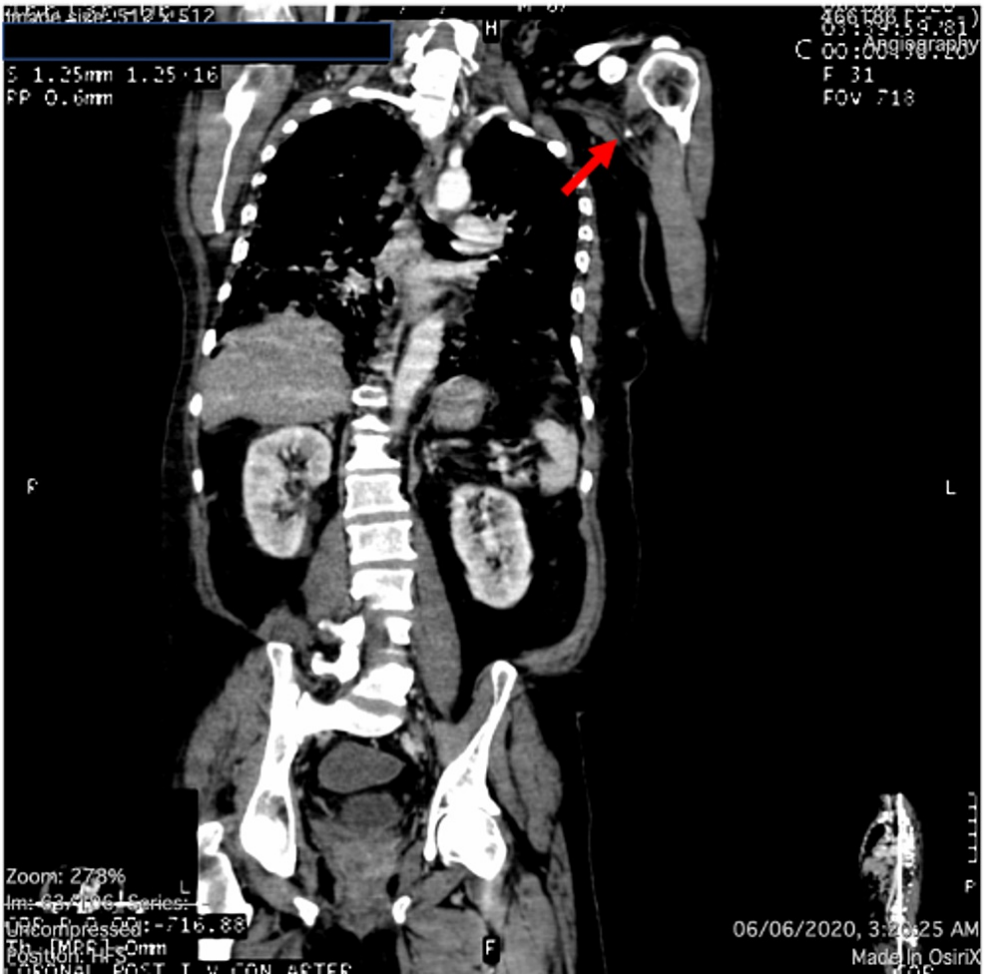
Coronal section of CT aortogram showing cut-off sign of the contrast at the level of junction between the left subclavian artery and the axillary artery (red arrow).

**Figure 3:**
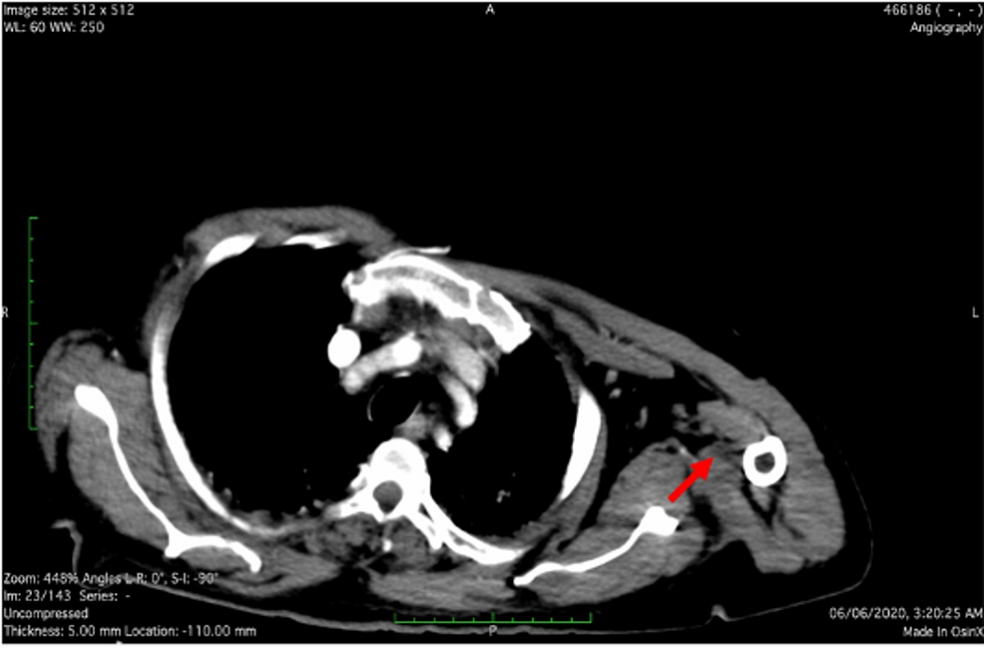
Axial section of CT aortogram showing filling defect within the left axillary artery (red arrow).

Upon initial assessment, he was found to have weak left radial pulse with warm limb and normal other peripheral pulses. Within 4 hours, the left radial pulse became absent, and the hand became cold. Urgent CT aortogram was performed, which showed cut-off sign of the contrast at the level of the junction between the left subclavian artery and the axillary artery, denoting occlusion (thrombosis) (Figures 2, 3).

**Figure 4:**
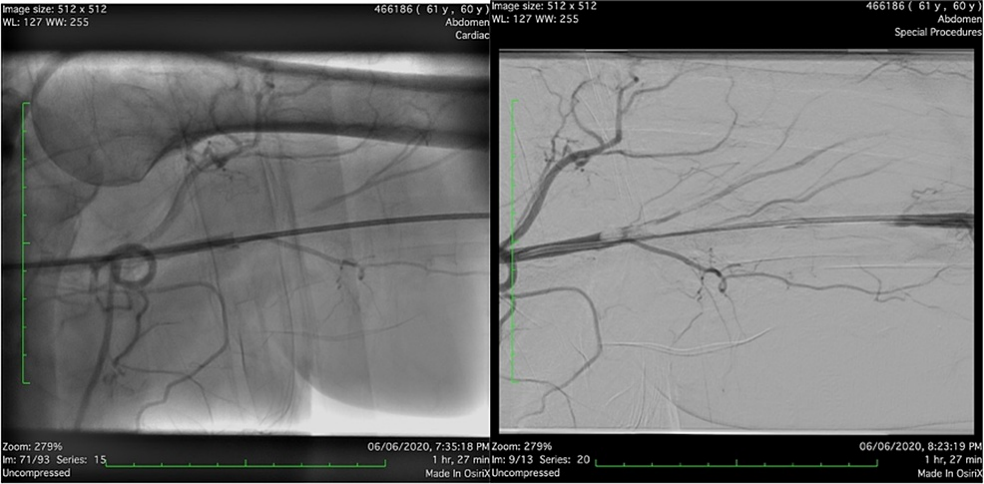
Pre-procedure angiography of the left brachial artery showing complete thrombosis extending from the axillary artery downward to the brachial artery.

**Figure 5:**
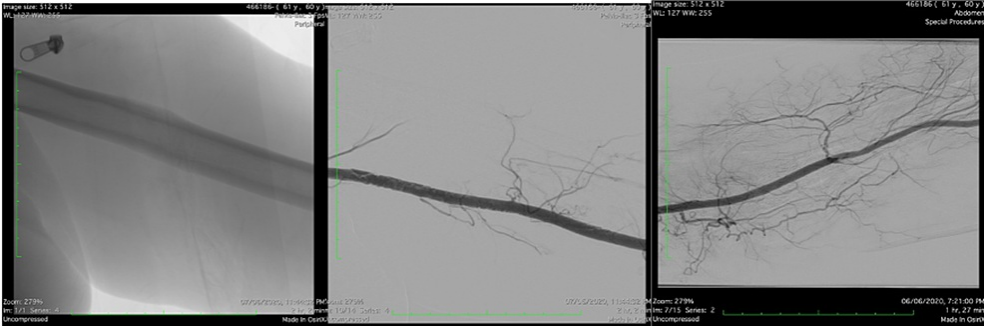
Post-procedure angiography of the left upper limb showing normal opacification with contrast from the axillary artery down to the brachial artery.

Work-up for embolic source was performed. Echocardiogram showed normal left ventricle systolic function with no evidence of intracardiac thrombus. Angioplasty was performed as an emergency procedure to save the limb by the interventional radiologist to retrieve the thrombus and resume perfusion followed by tissue plasminogen activator (TPA) infusion in the limb, but it failed. Therefore, brachial embolectomy was performed in the operating room with incision at the cubital fossa and embolectomy of the thrombus that extended from the brachial artery upward to the axillary artery. Post-procedure angiogram revealed patent brachial, axillary, subclavian, and ulnar arteries, and slow flow of radial artery (Figures 4, 5).

Heparin infusion was started post-procedure, with a target partial thromboplastin time (PTT) of 60-80 seconds, and clot was sent for histopathology. Full anticoagulation was continued until D-dimer levels normalized, and then it was switched to an SC prophylactic dose.

After two weeks, the patient’s COVID-19 PCR test became negative, but we were unable to liberate him from mechanical ventilation. Therefore, tracheostomy was performed and weaning trials were started. Unfortunately, after another two weeks, he developed ventilator-associated pneumonia (VAP) and septic shock that evolved to multiorgan failure and ended with death.

## Discussion

Thrombosis in critical care setting was described long time ago, but it mainly involved the lower limb as DVT [11] complicated by PE. The main risk factor for DVT in ICU was linked to immobility leading to blood stagnation, which has been tremendously reduced by DVT pharmacoprophylaxis. On the other hand, idiopathic arterial thrombosis is not frequent in a critical care setting as the mechanisms are a bit different than that of the venous ones and are usually iatrogenic or associated with invasive procedures [12,13]; arterial blood flow is usually fast and does not give chance for clot formation like venous ones [8,14,15].

Thrombosis in COVID-19 disease has been linked to multiple mechanisms, including endothelial cell damage through binding of viral spike protein to the angiotensin-converting enzyme 2 (ACE-2) receptor that is expressed mainly in the heart, lung, and intestine, and also found on the endothelium of all blood vessels. Once it is bound to the endothelial cells, it induces expression of intercellular adhesion molecules, attraction of macrophages that secrete collagenases degrading already found atherosclerotic plaque exposing a raw surface inviting clot-forming factors, and ending with endothelitis promoting in situ thrombosis [16]. Another mechanism is the inflammatory storm through secretion of mediators, such as interleukin-6 (IL-6), and production of C-reactive protein (CRP), fibrinogen, ferritin, and D-dimer, which triggers the complement cascade, thus inducing a hypercoagulable state [7]. All these mechanisms have been linked to the degree of COVID-19 severity [17]. Additionally, autoantibodies, such as antiphospholipid and anticardiolipin, have been described in sporadic cases of COVID-19; another theory to explain hypercoagulability is related to the neutrophil extracellular traps that are formed to contain the virus within vessels, which have been associated with triggering of the intrinsic coagulation pathway [18]. Blood stagnation inside vessels due to dysregulation of mediators that regulate local blood supply, including nitric oxide and endothelin 1, is another contributing factor, especially in pulmonary circulation [19]. These mechanisms are more pronounced in the lungs that are highly exposed to the virus and are in the nidus of the inflammatory storm, especially in the microcirculation, but they have been observed in other organs, including the heart, liver, intestine, and central nervous system.

Mechanisms of macrovascular thrombosis in COVID-19 are less understood as compared to microvascular involvement. Possible mechanisms that are usually observed in critically ill patients are high dose of vasopressors, iatrogenic arterial injury, or spontaneous arterial dissection. Furthermore, multiple records have shown a significant rise in the risk of macrovascular thrombosis in COVID-19 patients compared to non-COVID patients having the same cardiovascular risk profiles [20].

Thrombosis associated with COVID-19, whether venous or arterial, has been associated in multiple studies with increased adverse outcomes [10,21]. Still, studies reporting its effect on mortality are limited. Many predictive models were developed to predict the clinical outcome of COVID-19 patients, especially in the critical care setting, but did not include thrombosis, but risk factors for thrombosis, such as D-dimer, high CRP, fibrinogen, and other inflammatory markers, were predictive of poor outcome [22].

Our patient had cardiovascular risk in addition to severe COVID-19 from the beginning, as he required mechanical ventilation from the first day with high oxygen requirement and high markers of hypercoagulability. Thrombosis was evident on presentation, which makes iatrogenic cause less likely, and we did not find any embolic source [23]. As the patient’s arterial thrombosis was managed successfully, it is unlikely that it contributed to his final outcome, which was probably dependent on other established risk factors, namely his age, poor respiratory reserve due to underlying chronic obstructive pulmonary disease, comorbidities, and prolonged ICU stay with its complications, namely VAP and severe sepsis.

Thrombosis in the setting of COVID-19 has been described at various stages of the disease [23], either at presentation or even might be the presenting symptom, or discovered accidentally during the course of the disease; such variation could be related to disease severity on presentation, or progression of the disease severity during its course, or less likely to interventions by medical staff.

We emphasize the importance of monitoring COVID-19 patients for thrombotic events from the time of presentation, and frequently during their hospitalization, as they are at an increased risk, especially for patients with severe disease. It is challenging to diagnose those patients once they are sedated on mechanical ventilation due to limited verbal communication until florid signs appear.

Even though more than a year has elapsed since the announcement of the COVID-19 pandemic and its strong association with hypercoagulability causing clinical thrombosis, especially in severe cases, still we do not have evidence-based guidelines addressing anticoagulation in those patients. The International Society on Thrombosis and Haemostasis (ISTH) stated that increased D-dimer is significantly associated with high mortality and recommended only prophylactic anticoagulation with low molecular weight heparin (LMWH) for all hospitalized patients in the absence of contraindication [24]. Barret et al. in a commentary criticized the ISTH recommendations for being deficient and not meeting expectations based on his clinical observations of thrombosis in COVID-19 patients, especially those having high levels of D-dimer and fibrinogen, and recommended therapeutic level of anticoagulation with unfractionated heparin or LMWH in patients without significant bleeding risks [25]. Such controversy is fed by uncertain evidence in that regard. Lemos et al. has shown that full anticoagulation improves gas exchange and decreases the need for mechanical ventilation in severe COVID-19 [26], while the INSPIRATION study (intermediate versus standard-dose prophylactic anticoagulation and statin therapy versus placebo in critically-ill patients with COVID-19) preliminary analyses did not reduce a composite end point of venous or arterial thrombosis or death [27]. The COVID-19 Treatment Guidelines Panel and guideline panels of the American Society of Hematology and the American College of Chest Physicians as of February 2021 recommend treating all hospitalized patients with COVID-19, including critically ill patients, with prophylactic dose anticoagulation only so far.

## Conclusions

The COVID-19 pandemic is still evolving with new mutations and variants. It is a disease that carries a lot of medical mysteries and challenges, including marked hypercoagulability, especially in its severe form. Critical thrombotic events have been described extensively, especially in critically ill patients. Meticulous attention should be paid in that regard to discover them promptly. Prophylactic anticoagulation is the standard practice for all critically ill patients; however, for COVID-19 patients, they have to be evaluated thoroughly and frequently for the indication of prophylactic full anticoagulation as per the evolving guidelines to avoid potential disastrous thrombotic events.
